# Is distortion of the bioprosthesis ring a risk factor for early calcification ?

**DOI:** 10.1186/1749-8090-5-77

**Published:** 2010-10-07

**Authors:** Jose Manuel Martinez Cereijo, Jose Rubio Alvarez, Juan Sierra Quiroga, Anxo Martinez de Alegria, Jose Manuel Suarez Peñaranda

**Affiliations:** 1 Department of Cardiovascular Surgery, Santiago de Compostela University Hospital. La Choupana, Santiago de Compostela 15706, Spain; 2 Department of Radiology, Santiago de Compostela University Hospital. La Choupana, Santiago de Compostela 15706, Spain; 3 Department of Pathology, Santiago de Compostela University Hospital. La Choupana, Santiago de Compostela 15706, Spain

## Abstract

**Background:**

As the population ages, bioprosthesis are increasingly being used in cardiac valve replacement. Pericardial bioprosthesis combine an excellent hemodynamic performance with low thrombogenicity, but valve failure associated with calcification remains a concern with these valves. We describe distortion of the bioprosthesis ring as a risk factor for early calcification.

**Methods:**

A total of 510 patients over the age of 70 years underwent isolated aortic valve replacement with the Mitroflow (A12) pericardial bioprosthesis. Thirty two patients (6,2%) have undergone a second aortic valve replacement due to structural valve dysfunction resulting from valve calcification. In all patients a chest radiography and coronary angiography was performed before reoperation. A 64 Multidetector Computed Tomography (MDCT) with retrospective ECG gating study was performed in four patients to evaluate the aortic bioprosthesis.

**Results:**

Chest radiography showed in all patients an irregular bioprosthesis ring. At preoperative coronary angiography a distorted bioprosthesis ring was detected in all patients. Macroscopic findings of the explanted bioprostheses included extensive calcification in all specimens.

**Conclusion:**

There was a possible relationship between early bioprosthetic calcification and radiologic distortion of the bioprosthesis ring.

## 

As the population ages, bioprosthesis are increasingly being used in cardiac valve replacement. Pericardial bioprosthesis combine an excellent hemodynamic performance [1] with low thrombogenicity [2], but valve failure associated with calcification remains a concern with these valves [3]. We describe distortion of the bioprosthesis ring as a risk factor for early calcification.

## Materials and methods

A total of 510 patients over the age of 70 years underwent isolated aortic valve replacement with the Mitroflow (A12) pericardial bioprosthesis at our hospital. Sixty-five percent of patients undergoing aortic valve replacement received a 21 mm valve. Demographic data at time of reoperation are listed in Table 1 and the size of the bioprosthesis in Table 2 To date 32 patients (6,2%) have undergone a second aortic valve replacement due to structural valve dysfunction (SVD) resulting from valve calcification. The mean time between the first operation and reoperation was 70 months (range 36 to 98 months).

**Table 1:** Demographic data.

Age (range)	77 ± 4 (71 - 86)
Female/Male	18/14
Aortic Stenosis	28 (87,5%)
Renal insufficiency	0
Hypertension	14 (43,7%)
Diabetes	2 (6,25%)

**Table 2:** Bioprosthesis size.

Valve Size (mm)	At reoperation
19	1
21	20
23	10
25	1

In all patients a chest radiography and coronary angiography was performed before reoperation.

A 64 Multidetector Computed Tomography (MDCT) with retrospective ECG gating study was performed in four patients to evaluate the aortic bioprosthesis. In two patients the study was performed three weeks after aortic valve replacement with the Mitroflow A12 pericardial bioprosthesis. Both bioprosthesis were normal by echocardiographic study. Another two patients had bioprosthesis with SVD.

A Histopathologic analysis of the removed bioprosthesis was performed by one experienced pathologist.

## Results

Macroscopic findings of the explanted bioprostheses included extensive calcification in all specimens. In all cases, one leaflet was more calcified than the others; in our experience, the right coronary leaflet was commonly the most calcified (19/32. 59,3%).

In all patients, serum levels of cholesterol, triglycerides, lipoprotein A and calcium were within the normal range. No patients were treated with calcium supplementation.

**Figure 1:**
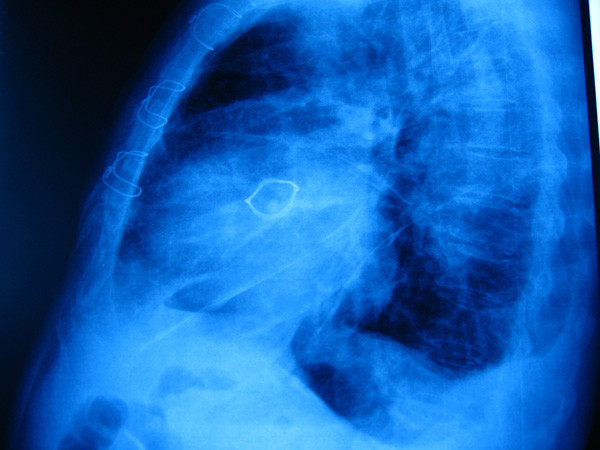
**Radiographic evaluation showed a misshapen bioprosthesis ring**.

**Figure 2:**
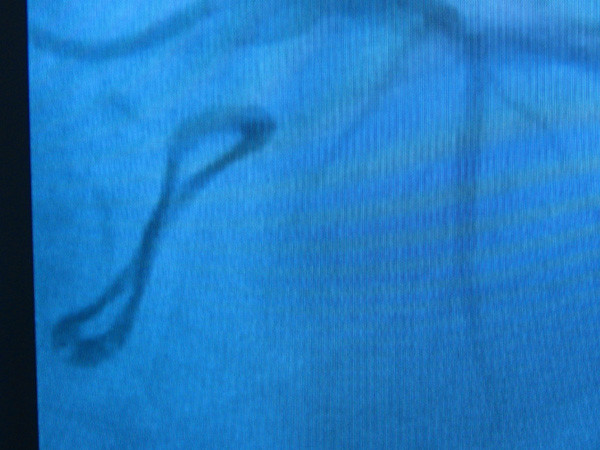
**Preoperative coronary angiography showed a misshapen bioprosthesis ring**.

**Figure 3:**
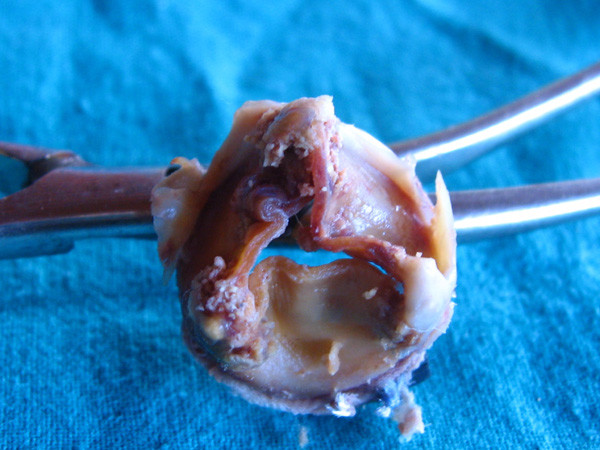
**Explanted bioprosthesis with leaflet distorted and calcification**.

Chest radiography (Figure 1) showed in all patients an irregular, non- circular bioprosthesis ring. At preoperative coronary angiography a distorted bioprosthesis ring was detected in all patients (Figure 2). An explanted bioprosthesis with leaflet distortion and right coronary leaflet calcification is shown in Figure 3.

**Figure 4:**
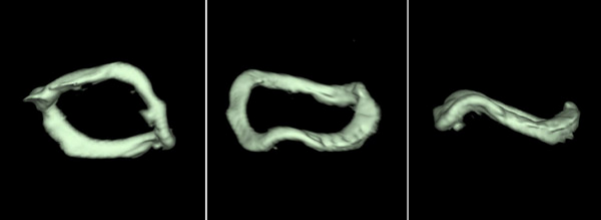
**Three different views of the bioprosthesis ring at MDCT**.

In two patients with normal bioprosthesis, the morphology of the leaflets and the degree of excursion were normal at MDCT, but a leaflet had a wave movement. The distorted ring was observed in these patients (Figure 4) and the same picture was observed in two patients with SVD.

The percentage of distortion of the bioprosthesis ring in patients without SVD was 76%, but no all patients had the same degree of distortion.

## Discussion

Bioprosthetic calcification is a multifactorial process; contributing factors include the type of implant used, mechanical stress, preservation method, patient age and technical correctness of the implantation procedure [4]. The rate of structural valve dysfunction is lower in elderly patients and early calcification in this group of patients is an uncommon event.

There is a strong relationship between mechanical stress and calcification in bioprosthesis [4,5]; in our study we noted that early calcification tended to develop in patients with radiologic distortion of the bioprosthesis ring. Liao and colleagues [4] observed similar results with bioprostheses removed from left ventricular assist devices. Distortion of the bioprosthesis ring may be due to technical problems at implantation [6] or compression around the ring. However we speculate that Dacron ring traction from the annular stitch may distort the normal planar geometry of Mitroflow pericardial bioprosthesis, leading to distortion of the pericardial leaflet mounted outside the stent and fixed to the Dacron ring, resulting in a higher mechanical stress.

In our patients other casual relationship was no found.

## Conclusion

In our experience there was a possible relationship between bioprosthetic calcification and radiologic distortion of the bioprosthesis ring.

## Authors' contributions

JMMC drafted the manuscript and did the patients follow-up.

JRA conceived of the study and participated in its design and coordination.

JSQ participated in the design of the study.

AMA carried out the radiologic studies.

JMSP carried out the pathologic studies.

All authors read and approved the final manuscript.

## Competing interests

The authors declare that they have no competing interests.
